# Molecular evidence of *Borrelia* spp. in bats from Córdoba Department, northwest Colombia

**DOI:** 10.1186/s13071-022-05614-y

**Published:** 2023-01-06

**Authors:** Yesica López, Sebastián Muñoz-Leal, Caty Martínez, Camilo Guzmán, Alfonso Calderón, Jairo Martínez, Ketty Galeano, Marina Muñoz, Juan David Ramírez, Álvaro A. Faccini-Martínez, Salim Mattar

**Affiliations:** 1 Instituto de Investigaciones Biológicas del Trópico, Universidad de Córdoba, Córdoba, Colombia; 2 Departamento de Ciencia Animal, Facultad de Ciencias Veterinarias, Universidad de Concepción, Chillán, Chile; 3 Centro de Investigaciones en Microbiología y Biotecnología-UR (CIMBIUR), Facultad de Ciencias Naturales, Universidad del Rosario, Bogotá, Colombia; 4 Molecular Microbiology Laboratory, Department of Pathology, Molecular and Cell-Based Medicine, Icahn School of Medicine at Mount Sinai, New York City, NY USA; 5 Research Institute, Fundación Universitaria de Ciencias de la Salud-FUCS, Bogotá, Colombia; 6 Servicios y Asesorías en Infectología-SAI, Bogotá, Colombia

**Keywords:** Bats, *Borrelia* sp., Ticks, Colombia

## Abstract

**Background:**

The genus *Borrelia* is composed of two well-defined monophyletic groups, the *Borrelia burgdorferi* sensu lato complex (Bb) and the relapsing fever (RF) group borreliae. Recently, a third group, associated with reptiles and echidnas, has been described. In general, RF group borreliae use rodents as reservoir hosts; although neotropical bats may also be involved as important hosts, with scarce knowledge regarding this association. The objective of this study was to detect the presence of *Borrelia* spp. DNA in bats from the department of Córdoba in northwest Colombia.

**Methods:**

During September 2020 and June 2021, 205 bats were captured in six municipalities of Córdoba department, Colombia. Specimens were identified using taxonomic keys and DNA was extracted from spleen samples. A *Borrelia*-specific real-time PCR was performed for the *16S rRNA* gene. Fragments of the *16S rRNA* and *flaB* genes were amplified in the positive samples by conventional PCR. The detected amplicons were sequenced by the Sanger method. Phylogenetic reconstruction was performed in IQ-TREE with maximum likelihood based on the substitution model TPM3+F+I+G4 with bootstrap values deduced from 1000 replicates.

**Results:**

Overall, 10.2% (21/205) of the samples were found positive by qPCR; of these, 81% (17/21) and 66.6% (14/21) amplified *16S rRNA* and *flaB* genes, respectively. qPCR-positive samples were then subjected to conventional nested and semi-nested PCR to amplify *16S rRNA* and *flaB* gene fragments. Nine positive samples for both genes were sequenced, and seven and six sequences were of good quality for the *16S rRNA* and *flaB* genes, respectively. The DNA of *Borrelia* spp. was detected in the insectivorous and fruit bats *Artibeus lituratus*, *Carollia perspicillata*, *Glossophaga soricina*, *Phyllostomus discolor*, and *Uroderma* sp. The *16S rRNA* gene sequences showed 97.66–98.47% identity with “*Borrelia* sp. clone Omi3,” “*Borrelia* sp. RT1S,” and *Borrelia* sp. 2374; the closest identities for the *flaB* gene were 94.02–98.04% with “*Borrelia* sp. Macaregua.” For the *16S rRNA* gene, the phylogenetic analysis showed a grouping with “*Candidatus* Borrelia ivorensis” and “*Ca*. Borrelia africana,” and for the *flaB* gene showed a grouping with *Borrelia* sp. Macaregua and *Borrelia* sp. Potiretama. The pathogenic role of the *Borrelia* detected in this study is unknown.

**Conclusions:**

We describe the first molecular evidence of *Borrelia* spp. in the department of Córdoba, Colombia, highlighting that several bat species harbor *Borrelia* spirochetes.

**Graphical Abstract:**

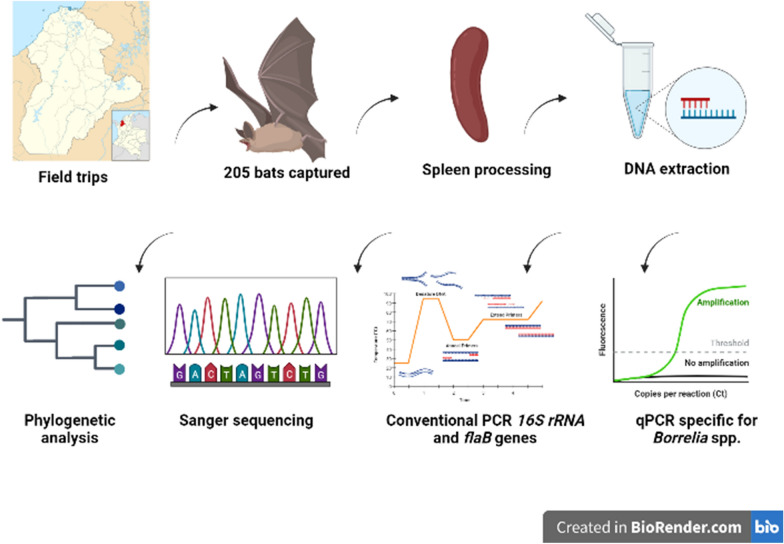

## Background

Pathogenic species of the genus *Borrelia* are zoonotic bacteria that cause emerging and re-emerging infectious diseases worldwide and constitute a major threat to public health [1]. This genus is composed of two well-defined monophyletic groups: the *Borrelia burgdorferi* sensu lato (Bb) complex, which cause Lyme borreliosis and are transmitted by hard ticks of the genus *Ixodes*, and borreliae of the relapsing fever (RF) group, transmitted mainly by soft ticks of the genus *Ornithodoros*, some species by ixodid ticks (e.g., *Borrelia miyamotoi*), and *Borrelia recurrentis*, transmitted by the clothing louse [2]. Recently, a third group associated with reptiles and echidnas (*Tachyglossus aculeatus*) has been described [3, 4], and share a common ancestor with the RF clade [3, 4].

Most species of *Borrelia* have complex transmission cycles interacting with multiple vertebrate hosts and vector ticks [1]. For instance, several members of the order Rodentia are reservoir hosts [5, 6]. Moreover, bats may also be involved as alternative hosts [7–12]. Indeed, several studies have reported *Borrelia* spp. in ticks collected from different bat species [13–20]. Interestingly, new putative taxa of *Borrelia* spirochetes, namely *Borrelia* sp. Macaregua and *Borrelia* sp. Potiretama, were detected in bats roosting in caves from Colombia and Brazil, respectively [10, 21].

Given their intimate relationship with emerging microorganisms that cause serious infections in humans, such as severe acute respiratory syndrome (SARS), Middle East respiratory syndrome (MERS) [22], and severe acute respiratory syndrome coronavirus 2 (SARS-CoV-2) [23], bats have recently been the center of attention for scientists. A remarkable fact is that bats harbor more viruses than rodents [24]. However, bacteria harbored by these mammals are neglected, even though they can represent zoonotic pathogens [25]. For example, RF group spirochetes such as *Ca.* Borrelia fainii and *Ca.* Borrelia johnsonii are among those neglected agents [26, 27]. In order to contribute to the understanding of *Borrelia* spp. associated with chiropterans, we carried out a prospective study to detect DNA of *Borrelia* in organs of bats derived from a COVID-19 study, collected in the department of Córdoba, Colombia.

## Methods

### Study area and bat captures

**Fig. 1:**
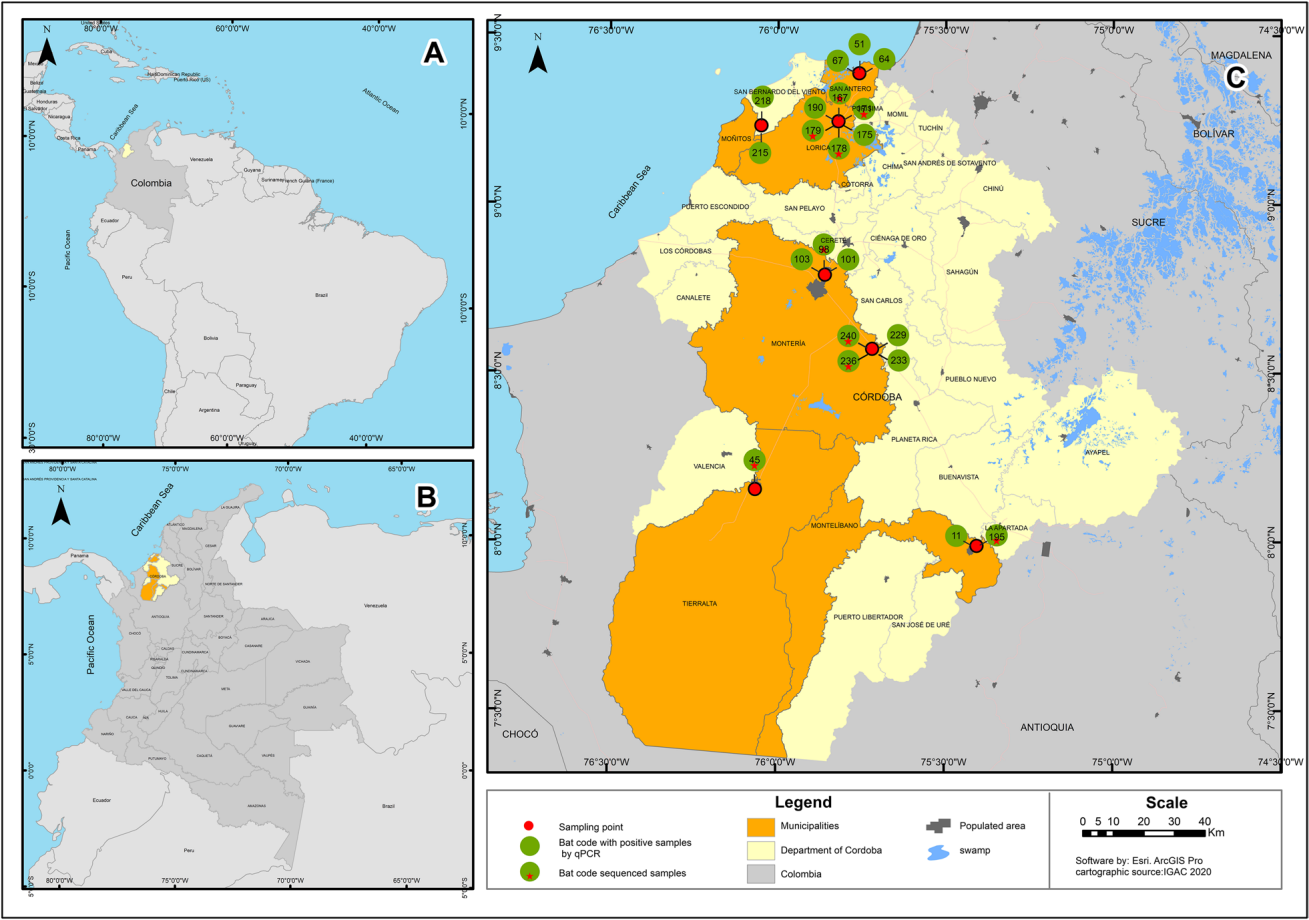
**A** Map of South America showing the location of Córdoba Department within Colombia. **B** Map of Córdoba Department showing the investigated municipalities. **C** Sampled municipalities in the department of Córdoba showing the collection sites of real-time PCR-positive bats

Field trips were carried out during September 2020 and June 2021 in six municipalities of Córdoba Department (Montelíbano, Tierralta, San Antero, Montería, Lorica, and Moñitos) in urban, peri-urban and rural areas with similar environmental conditions: altitude of 12–87 m, 25–28 °C, and average relative humidity of 81% (Fig. 1; Table 2).

Bats were captured using mist nets (6 m × 2 m). Males and females without signs of pregnancy were included in the study. Captured bats were euthanized with an overdose of sodium pentobarbital (200 mg) at a dose of 0.05 mg/g [28]. Following taxonomic identification using dichotomous keys [29], spleen samples were extracted, stored in sterile tubes, and kept at −80 °C until processing. The capture of bats was carried out under the permits of the National Environmental Licensing Authority (ANLA), Resolution No. 00914. All the procedures were approved by the ethics committee of the Faculty of Veterinary Medicine and Zootechnics of the Universidad de Córdoba (No. 003 of December 6, 2019).

### Molecular and phylogenetic analyses

DNA extraction was performed in spleen samples using the GeneJET Genomic DNA Purification Kit (Thermo Scientific) following the manufacturer's instructions. A conventional polymerase chain reaction (cPCR) targeting the mammalian *ß-actin* gene was performed as an internal control for each extraction [30]. Positive samples were then submitted to a real-time PCR (qPCR) specific for the genus *Borrelia*, [31]. Samples with cycle threshold values ≤ 33 were considered positive and then subjected to conventional nested and semi-nested PCRs to amplify *16S rRNA* and *flaB* genes fragments [32, 33]. The annealing temperature for the *16S rRNA* gene in the cPCR was modified in the first round (FD3 [f]/T50 [r]) and second round (Rec4 [f]/Rec9 [r]) from 56 °C to 54 °C (Table 1). Genomic DNA of *Borrelia anserina* and molecular-grade water were used as positive and negative controls, respectively. Amplicons of the expected size were Sanger-sequenced at Macrogen (Seoul, Korea), sequences assembled with Geneious, and the consensuses were compared in GenBank using BLASTn [34].

**Table 1:** Primers used to amplify *Borrelia* genes in this study

Gene	Round	Primer name	Sequence 5′–3′	Temp [°C]	Base pairs
*16S rRNA* qPCR [31]		Bor16S3F	AGCCTTTAAAGCTTCGCTTGTAG	60	148
		Bor16S3R	GCCTCCCGTAGGAGTCTGG		
		Probe Bor16S3P	[6FAM] CCGGCCTGAGAGGGTGAACGG		
*16S rRNA* [32]	First round	FD3 [f]	AGAGTTTGATCCTGGCTTAG	54	1489
		T50 [r]	GTTACGACTTCACCCTCCT		
	Second round	FD3 [f]	AGAGTTTGATCCTGGCTTAG	56	730
		16s-1 [r]	TAGAAGTTCGCCTTCGCCTCTG		
	Second round	16s-2 [f]	TACAGGTGCTGCATGGTTGTCG	56	462
		T50 [r]	GTTACGACTTCACCCTCCT		
	Second round	Rec4 [f]	ATGCTAGAAACTGCATGA	54	520
		Rec9 [r]	TCGTCTGAGTCCCCATCT		
*flab* [33]	First round	FlaRL [f]	GCAATCATAGCCATTGCAGATTGT	55	665
		FlaLL [r]	ACATATTCAGATGCAGACAGAGGT		
	Second round	FLaRS [f]	CTTTGATCACTTATCATTCTAATAGC	55	491
		FlaLL [r]	ACATATTCAGATGCAGACAGAGGT		
	Second round	FlaRL [f]	GCAATCATAGCCATTGCAGATTGT	55	528
		FLaLS [r]	AACAGCTGAAGAGCTTGGAATG		

The alignments were built in Clustal Omega [35]. For the *16S rRNA* and the *flaB* gene, 70 and 81 sequences downloaded from GenBank were used, respectively [36]. Phylogenetic reconstructions were performed in IQ-TREE with the maximum likelihood method using the TPM3+F+I+G4 nucleotide substitution model with 1000 bootstraps. Trees were visualized and edited with iTOL v5 [37]. *Brachyspira pilosicoli* was used as root.

## Results

Two hundred and five spleen samples from different bat species were processed. Amplicons of the expected size for the *ß-actin* gene were obtained in all samples, thus confirming successful DNA extraction. Overall, 10.2% (21/205) were positive for the *Borrelia* *16S rRNA* gene by qPCR. In 81% (17/21) and 66,6% (14/21) positive samples, it was possible to amplify *16S rRNA* and flaB genes by cPCR. Regarding bat species, *Borrelia* DNA was detected in 5.4% (11/21) of specimens in the genus *Phyllostomus*, 2.4% (5/21) in *Glossophaga*, 1.5% (3/21) in *Carollia*, 0.5% (1/21) in the genus *Artibeus*, and 0.5% (1/21) in *Uroderma*. For each *Borrelia* gene, nine randomly selected cPCR products were sequenced; however, good quality sequences were obtained in seven *16S rRNA* and six *flaB* samples. Most identical sequences after Blastn comparisons for the *16S rRNA* gene matched spirochetes detected in *Ornithodoros mimon* from Brazil [38]; a *Borrelia* sp. reported in *Amblyomma varanense* from *Varanus salvator* (reptile) from Indonesia [39]; and a *Borrelia* sp. reported in hard ticks from Portugal [40]. For the *flaB* gene, sequences were most identical with “*Borrelia* sp. Macaregua” haplotypes, detected in bats from the Macaregua cave in Santander, Colombia [10] (Table 2).

**Table 2:** Detection of *Borrelia* DNA in bats

Bat code	Municipality	Longitude	Latitude	qPCR-positive bat species	Sex	*16S rRNA* sequence identity (GenBank code)	*flaB* sequence identity (GenBank code)
11	Montelíbano	75°24′15.02′′	7°59′13.67′′	*Carollia perspicillata*	Male	–	–
45	Tierralta	76° 3′9.48′′	8° 9′15.74′′	*Carollia perspicillata*	Male	–	98.04% *Borrelia* sp. Macaregua122 (MT154618.1)
51	San Antero	75°45′29.15′′	9°23′9.30′′	*Phyllostomus discolor*	Male	–	–
64	San Antero	75°45′29.15′′	9°23′9.30′′	*Artibeus lituratus*	Male	–	–
67	San Antero	75°45′29.15′′	9°23′9.30′′	*Phyllostomus discolor*	Male	–	–
98	Montería	75°51′29.51′′	8°47′19.82′′	*Phyllostomus discolor*	Female	98.15% *Borrelia* sp. Omi3MT (MT013212.1)	96.64% *Borrelia* sp. Macaregua122 (MT154618.1)
101	Montería	75°51′29.51′′	8°47′19.82′′	*Phyllostomus discolor*	Male	–	–
103	Montería	75°51′29.51′′	8°47′19.82′′	*Phyllostomus discolor*	Male	–	–
167	Lorica	75°49′10.69′′	9°14′36.93′′	*Glossophaga soricina*	Male	97.90% *Borrelia* sp. Omi3MT-(MT013212.1)	–
171	Lorica	75°49′10.69′′	9°14′36.93′′	*Glossophaga soricina*	Male	–	–
175	Lorica	75°49′10.69′′	9°14′36.93′′	*Glossophaga soricina*	Male	–	–
178	Lorica	75°49′10.69′′	9°14′36.93′′	*Glossophaga soricina*	Male	98.47% *Borrelia* sp. Omi3MT (MT013212.1)	–
179	Lorica	75°49′10.69′′	9°14′36.93′′	*Phyllostomus discolor*	Female	98.05% *Borrelia* sp. Omi3MT (MT013212.1)	95.80% *Borrelia* sp. Macaregua122 (MT154618.1)
190	Lorica	75°49′10.69′′	9°14′36.93′′	*Glossophaga soricina*	Male	–	–
195	Montelíbano	75°24′15.02′′	7°59′13.67′′	*Uroderma* sp.	Male	97.69% *Borrelia* sp. RT1S (LC428383.1)	95.94% *Borrelia* sp. Macaregua122 (MT154618.1)
215	Moñitos	76°05′21′′	9°15′13′′	*Phyllostomus hastatus*	Male	–	–
218	Moñitos	76°05′21′′	9°15′13′′	*Phyllostomus discolor*	Male	–	–
229	Montería	75°43′02′′	8°34′12′′	*Carollia perspicillata*	Male	–	–
233	Montería	75°43′02′′	8°34′12′′	*Phyllostomus discolor*	Female	–	–
236	Montería	75°43′02′′	8°34′12′′	*Phyllostomus discolor*	Male	97.66% *Borrelia* sp. 2374 (KT364304.1)	94.44% *Borrelia* sp. Macaregua122 (MT154618.1)
240	Montería	75°43′02′′	8°34′12′′	*Phyllostomus discolor*	Female	97.66% *Borrelia* sp. 2374 (KT364304.1)	94.02% *Borrelia* sp. Macaregua122 (MT154618.1)

**Fig. 2:**
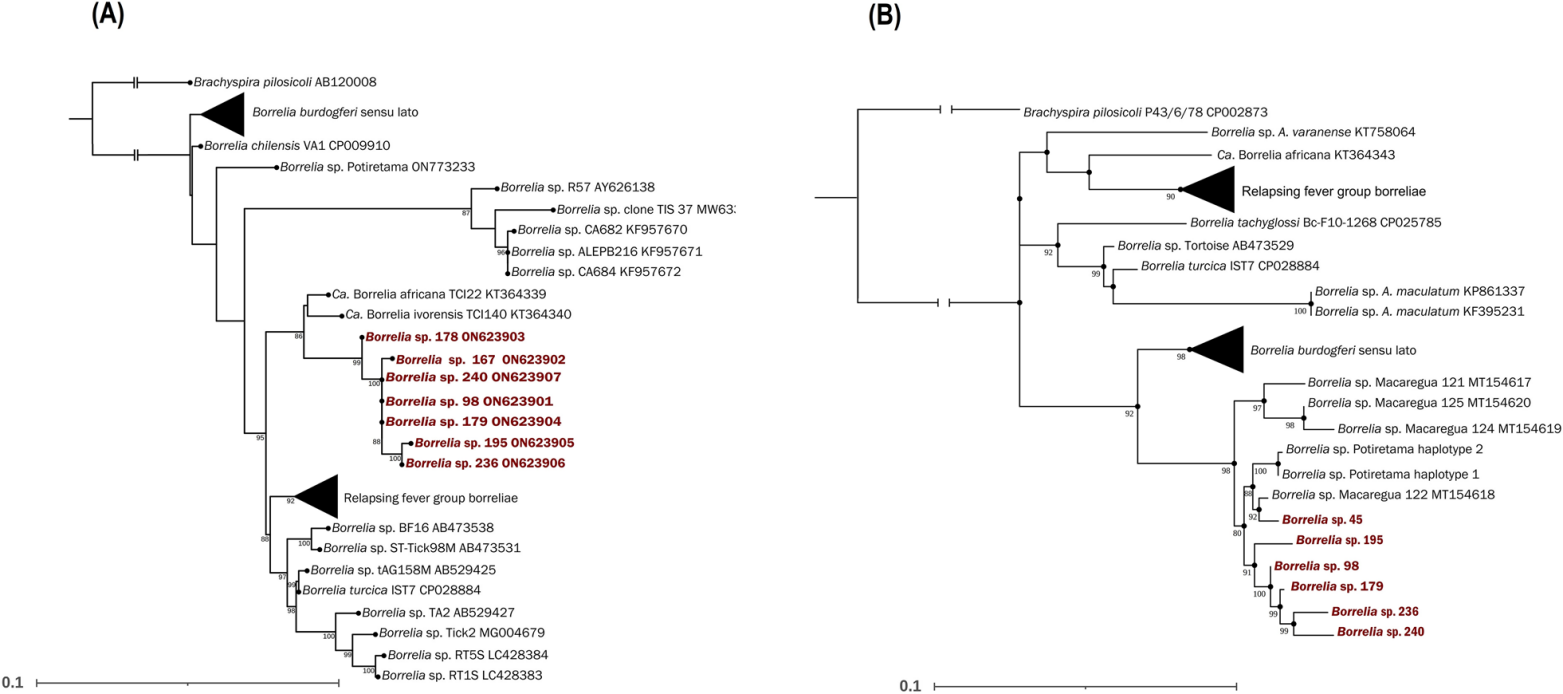
Phylogenetic analysis performed in this study. Trees are drawn to scale, with the scale bar indicating nucleotide substitutions per site. The position of the detected *Borrelia* spp. is highlighted in red. **A** Phylogenetic tree of *16S rRNA* gene constructed with 78 sequences. **B** Phylogenetic tree of the **flaB** gene built with 88 sequences

In the *16S rRNA* phylogeny, all the obtained sequences were grouped monophyletically and as a sister lineage to “*Candidatus* Borrelia ivorensis” and “*Candidatus* Borrelia africana” out of the RF or Lyme groups (Fig. 2A). The *flaB* phylogenetic tree places all our sequences into a monophyletic clade together with haplotypes of “*Borrelia* sp. Macaregua” and *Borrelia* sp. Potiretama (Fig. 2B).

## Discussion

To date, there are few studies that show the presence of *Borrelia* in bats. One of the first reports was made by Nicolle and Comte in 1905, with the finding of spirochetes in the blood of *Vespertilio kuhli* from Tunisia [41]. Then, in 1945, Nájera Angulo demonstrated the susceptibility of four bats species (*Miniopterus schreibersii*, *Myotis myotis*, *Rhinolophus euryale*, and *Rhinolophus hipposideros minimus*) from Spain to a “Hispanic spirochete” after inoculation of blood from infected guinea pig [42].

Later, in 2006, in the United States, through serological tests it was possible to verify the circulation of borreliae in *Eptesicus fuscus* bats [17]. Subsequently, in 2009, in England, spirochetes were identified in liver tissue stained with Warthin–Starry in a bat of the genus *Pipistrellus*, and it was confirmed as an RF group *Borrelia*, obtaining a 776-base-pair (bp) segment of the *flaB* gene [7].

In recent years, *Ca.* Borrelia fainii was reported in *Myotis* sp. [9], *Rhinolophus pusillus*, and *Myotis davidii* bats in China [12]. Regarding Latin America, in Mexico, Colunga-Salas et al. detected two new lineages of *Borrelia*, one RF and one Bb, in *Saccopteryx bilineata*, *Choeroniscus godmani*, *Sturnira parvidens*, and *Lasiurus cinereus* [11]. In Colombia, in 1968, Marinkelle and Grose observed spirochetes in a blood smear of *Natalus tumidirostris* [43] from the Macaregua cave, and in 2020 Muñoz-Leal et al. detected a new putative taxon within the genus *Borrelia* in *Carollia perspicillata* captured in the same cave [10]. Likewise, in 2022, Jorge et al. detected *Borrelia* sp. Potiretama in *Desmodus rotundus* bats, in the municipality of Potiretama in Brazil [21]. In the current study, we detected *Borrelia* DNA in five species of bats (*C. perspicillata*, *Phyllostomus discolor*, *Artibeus lituratus*, *Glossophaga soricina*, and *Uroderma* sp.) captured in Colombia, reinforcing the fact that bats do harbor *Borrelia* spirochetes.

In our study, two phylogenetic analyses were performed including *Borrelia* spp. previously detected in bats from South America, such as *Borrelia* sp. Macaregua and *Borrelia* sp. Potiretama [10, 21]. Unfortunately, *16S rRNA* sequences for *Borrelia* sp. Macaregua are not available in GenBank, so it was not possible to perform comparisons with this species using this gene. Interestingly, *16S rRNA* phylogeny depicts the group of *Borrelia* spp. detected in this study as paraphyletic regarding *Borrelia* sp. Potiretama reported in bats from Brazil, and *Ca.* Borrelia ivorensis and *Ca*. Borrelia africana, two species detected in African ticks, as closely related. The fact that some South American borreliae are phylogenetically closer to their African representatives has also been observed for spirochetes characterized in soft ticks from Brazil and Chile [38, 44]. However, any conclusion regarding the phylogenetic position of the *Borrelia* spp. detected in our study is premature, since we obtained short *16S rRNA* gene sequences. Indeed, the fact that the *flaB* tree showed the *Borrelia* sequences of our study forming a monophyletic group with *Borrelia* sp. Potiretama, contradicting the topology of the *16S rRNA* tree, could also be explained by the limited data submitted to analysis for this gene (381–435 bp).

A monophyletic group of *Borrelia* spp. associated with neotropical bats roosting in caves has been recently proposed based primarily on phylogenetic analyses of *flaB* sequences [10, 21]. Our study supports this hypothesis, adding more genovariants to this group of bat species inhabiting wooded areas. Because of their defense-immune tolerance capacities, bats are considered excellent reservoirs that favor the emergence of novel viruses [45]. The reasons that South American bats seem to harbor such a remarkable diversity of *Borrelia* haplotypes could relate to their immunological system as well.

Finally, isolating *Borrelia* spp. circulating in bats and sequencing their genomes should now be the focus in order to clearly elucidate the phylogenetic relationships. The study of *Borrelia* in bats is important because several species associated with these mammals, such as *Ca.* Borrelia fainii and *Ca.* Borrelia johnsonii, have recently been detected in humans in Africa and the United States, respectively [9, 12, 26, 27]. Therefore, the pathogenic roles of spirochetes detected in neotropical bats should be further investigated.

## Data Availability

All data generated or analyzed during this study are included in this published article.

## Abbreviations

Bb: *Borrelia burgdorferi* sensu lato
RF: Borreliae of the relapsing fever group
